# Predictors of Radiation Exposure in Transcatheter Aortic Valve Replacement Procedures in a Large Hospital System

**DOI:** 10.1016/j.jscai.2026.104386

**Published:** 2026-03-24

**Authors:** Logan L. Vincent, Michael Simanonok, Kateri J. Spinelli

**Affiliations:** Center for Cardiovascular Analytics, Research and Data Science (CARDS), Providence Heart Institute, Providence Research Network, Portland, Oregon

**Keywords:** cumulative air kerma, fluoroscopy time, radiation dose area product, sex disparities in subspecialty training, transcatheter aortic valve replacement

Radiation exposure is a necessary but modifiable element of interventional cardiology cases. Given its effect on patient safety and long-term operator wellbeing, our understanding of radiation exposure must consider current case types and procedural environments. With the rapid evolution and rise of structural heart interventions, particularly with increasing rates of transcatheter aortic valve replacement (TAVR), understanding procedure-specific variables that affect radiation safety is essential for protecting both operators and patients.

Females are underrepresented in the field of cardiology, with even lower female representation within procedural subspecialties.^1^ Only 4.1% of interventional cardiologists are female, and 3.6% of TAVR operators self-identify as women.^2^^,^^3^ Although there are many determinants of career choice, concerns about radiation exposure are particularly impactful for female cardiologists considering procedural subspecialties.^4^^,^^5^ Within this context, we sought to understand determinants of radiation exposure during TAVR to identify potential for exposure reduction and shed light on real-world radiation exposure to help educate physicians as they make choices about the pursuit of subspecialty training.

We performed a retrospective cohort analysis of TAVR patients ≥18 years old within the Providence St. Joseph Health System from January 1, 2018, through December 31, 2022. We analyzed data that were collected according to the Society of Thoracic Surgeons (STS)/ACC Transcatheter Valve Therapy (TVT) Registry specifications.^6^ Patients were excluded if the TAVR procedure was aborted for any reason, or if procedure location data were missing (ie, facility, procedure room type). We added 1 non-TVT variable of operator experience, defined as the time in years between completion of medical school and the procedure date. This study was approved by the Providence St. Joseph Health institutional review board, with a waiver of informed consent.

Our aim was to examine correlations between patient characteristics, clinical factors, facility features, operator experience, and radiation exposure outcomes (fluoroscopy time, dose area product [DAP], and cumulative air kerma [CAK]). Linear mixed effects models were used to test the effect of patient and procedure-related factors on the 3 primary outcomes. Response variables were log-transformed for analysis because of scale and skewness; reported estimates were back-transformed. To account for variability across facilities and operators, we included a random effect term for operator (N = 92) nested within facility (N = 15); 7 operators performed TAVR procedures at multiple facilities. For fixed effects in our models, we included patient age, sex (male/female), body mass index (BMI), STS risk score, estimated glomerular filtration rate (eGFR), procedure year, procedure status (elective vs urgent, emergency, and salvage), valve in valve (Y/N), TVT access site (femoral vs other), embolic protection device (Y/N), device type (balloon vs self-expanding), room type (cardiac catheter lab vs hybrid), and operator experience. Missing data rates were <3% for all variables included in analyses, with the exceptions of DAP (11.9%) and CAK (5.3%). Patients with missing values for these outcomes were excluded from the respective models. All analyses were performed in R version 4.3.2 (R Core Team 2023).

The study included 8976 TAVR procedures (mean age 79.61 ± 8.4 years, 42.2% female) performed by 92 operators across 15 hospitals (Supplemental Table S1). The STS risk score was 4.4, 44% and 34% of patients had stage 2 and stage 3 chronic kidney disease, respectively, and the average eGFR was 66.7 mL/min/1.73 m^2^. Most procedures were elective (91%), with femoral access (95.5%) and self-expanding valves (71.2%) (Supplemental Table S2). Seventy-five percent were performed in a hybrid room, and 25% were performed in a cardiac catheterization lab. Median fluoroscopy time was 12.0 minutes, CAK was 503 mGy, and DAP was 57,000 mGy·cm^2^.

Cardiac catheterization labs had 8% lower CAK compared to hybrid operating rooms (Figure 1). Embolic protection devices increased fluoroscopy time by 41% (∼7.1 minutes) and CAK by 15%. Femoral access reduced fluoroscopy time by 21% (∼4.8 minutes). Valve-in-valve procedures increased fluoroscopy time by 29% (∼5.2 minutes) and CAK by 23%. Elective procedures had 3% less fluoroscopy time and 9% less CAK. Fluoroscopy time, DAP, and CAK were 4%, 8%, and 2% greater, respectively, for every 5% increase in STS risk. BMI also increased DAP and CAK by 10% and 12% for every 5 units of BMI. DAP and CAK decreased with patient age by 4% every 5 years. Female patients received 29% less DAP and 28% less CAK. Every 10-unit increase in eGFR increased fluoroscopy time by 0.04%. Fluoroscopy time and CAK decreased by 4% and 10% per year over the study time period, while DAP increased by 3% per year. Self-expanding devices increased fluoroscopy time by 8% (∼1.5 minutes) and CAK by 12%, presumably due to the need for slow deployment, potential recapture/reposition, and examination in multiple fluoroscopic angles. We observed no effect of operator experience on any radiation metrics.

**Figure 1:**
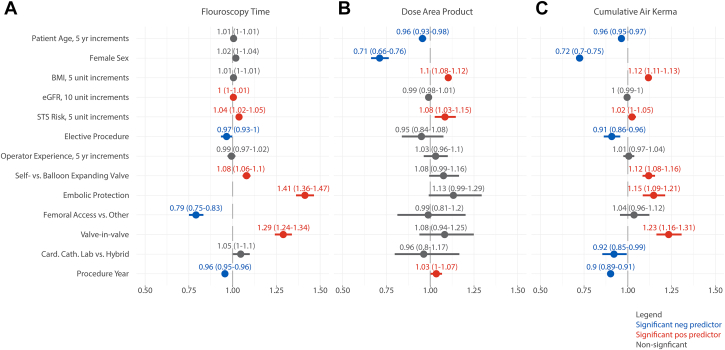
**Risk-adjusted predictors of radiation exposure during transcatheter aortic valve replacement.** Radiation exposure outcomes were (A) fluoroscopy time, (B) dose area product, and (C) cumulative air kerma. Reported estimates and 95% CIs are displayed. Blue font indicates statistically significant negative predictors, red font indicates statistically significant positive predictors, and gray font indicates odds ratios that were not statistically significant. BMI, body mass index; eGFR, estimated glomerular filtration rate; STS, Society of Thoracic Surgeons.

Our results show that there are a modest number of patient, procedural, and device factors that affect radiation exposure. However, the respective radiation increases attributable to each of these factors were quite small. Although these factors are important to be aware of to facilitate exposure reduction, both operators and patients can be reassured that radiation doses are not dramatically changed by any individual patient, procedural, or device factors. Notably, years of operator experience and room type (hybrid operating suite, cardiovascular operating room, or cardiovascular catheterization laboratory) did not significantly affect radiation dose.

Overall radiation doses have decreased over time within the study period. We suspect this is related to increased operator experience with the technology, evolution of delivery platforms and protocols to increase efficiency, the predominance of transfemoral access, and expansion of TAVR to lower-risk patients. Optimistically, some of the improvements in radiation outcomes we observed may be attributable to prioritization by operators of reducing cumulative radiation exposure. Study limitations include the retrospective design and limitations of the TVT registry data, which exclude information about procedure equipment (age of equipment, newer equipment models) and radiation safety protocols (lead aprons, eyeglasses, radiation shielding devices, etc). This study did not assess radiation exposure at the operator level; it assessed radiation exposure only at the patient level. Radiation safety tools and techniques were at the discretion of the hospital and operator and likely varied between the 15 facilities included in the study.

In conclusion, radiation exposure during TAVR remains a modifiable variable, with several factors each contributing small amounts to changes in radiation dose. Overall radiation doses were low, which can reassure female providers who are considering a career in structural interventions. Further prospective analyses are necessary to understand how operator awareness of these factors can lead to potential dose modification, as well as how new radiation safety technologies are affected by additional operator protections.

## Footnotes

To access the supplementary material accompanying this article, visit the online version of the *Journal of the Society for Cardiovascular Angiography & Interventions* at 10.1016/j.jscai.2026.104386.
