# Supplementary material for: Predictors of Radiation Exposure in Transcatheter Aortic Valve Replacement Procedures in a Large Hospital System
Source: J Soc Cardiovasc Angiogr Interv. 2026 Mar 24;5(4):104386. doi: 10.1016/j.jscai.2026.104386 (PMC13154614; doi:10.1016/j.jscai.2026.104386)
Supplement: Supplementary Data [file mmc1.docx]

***Journal of the Society for Cardiovascular Angiography***

**Predictors of Radiation Exposure in Transcatheter Aortic Valve Replacement Procedures in a Large Hospital System**

Logan L. Vincent, MD^1^, Michael Simanonok, PhD^1^, Kateri J. Spinelli, PhD^1^

1. Center for Cardiovascular Analytics, Research and Data Science (CARDS), Providence Heart Institute, Providence Research Network, Portland, Oregon

Corresponding Author: Logan Vincent, MD
Email: [logan.vincent@providence.org](mailto:logan.vincent@providence.org)

Address: 9427 SW Barnes Rd, Portland, OR 97225

Business phone: 503-216-1234 / Fax: 503-216-0920

Supplemental Table S1: Patient Demographics

| **Variables** | **All patients (N = 8976)** |
| --- | --- |
| Age | 79.61 ± 8.4 |
| Sex, female | 3787 (42.2%) |
| BMI | 29.4 ± 9.46 |
| Ethnicity, Hispanic | 319 (3.6%) |
| Race |  |
| American Indian | 118 (1.3%) |
| Asian | 173 (1.9%) |
| Black | 83 (0.9%) |
| Native Hawaiian/Pacific Island | 22 (0.30%) |
| White | 8413 (93.73%) |
| Other | 183 (2.0%) |
| STS risk score, % | 4.38 ± 3.69 |
| eGFR (mL/min/1.73m²) | 66.66 ± 25.7 |
| Chronic kidney disease |  |
| Stage 1 | 1394 (15.6%) |
| Stage 2 | 3948 (44.2%) |
| Stage 3 | 3026 (33.9%) |
| Stage 4 | 346 (3.9%) |
| Stage 5 | 223 (2.5%) |
| Diabetes | 3123 (34.8%) |
| Heart failure | 2935 (77.0%) |
| Hypertension | 7841 (87.4%) |
| Previous MI | 1705 (19.0%) |
| Peripheral artery disease | 1847 (20.6%) |
| Procedure history |  |
| CVA | 998 (11.1%) |
| CABG | 1170 (13.0%) |
| TAVR | 41 (0.96%) |

Data presented as n (%) of patients or mean ± SD

Abbreviations: BMI = body mass index, CABG = coronary artery bypass grafting, CVA = cerebrovascular accident, eGFR = estimated Glomerular Filtration Rate, MI = myocardial infarction, STS = society of thoracic surgeons, TAVR = transcatheter aortic valve replacement

Supplemental Table S2: Procedural Characteristics

| **Variable** | **All patients (N = 8976)** |
| --- | --- |
| Fluoroscopy time, minutes | 12.0 (8.9, 16.5) |
| Cumulative air kerma, mGy | 503 (280, 879) |
| Dose area product, mGy·cm² | 57000 (24540, 114556) |
| Room type |  |
| Hybrid* | 6710 (74.8%) |
| Cardiac catheterization lab | 2266 (25.3%) |
| Procedure status |  |
| Elective | 8168 (91.0%) |
| Urgent / Emergency / Salvage | 805 (9.0%) |
| Access site |  |
| Femoral | 8569 (95.5%) |
| Non-femoral | 405 (4.5%) |
| Valve in Valve | 574 (6.4%) |
| Embolic protection device | 945 (10.5%) |
| Valve type |  |
| Balloon expanding | 2587 (28.8%) |
| Self-expanding | 6389 (71.2%) |
| Year of procedure |  |
| 2018 | 1501 (16.7%) |
| 2019 | 1865 (20.8%) |
| 2020 | 1806 (20.1%) |
| 2021 | 1834 (20.4%) |
| 2022 | 1970 (22.0%) |

Data presented as n (%) of patients or median (IQR) 
* Hybrid = hybrid catheterization lab suite, hybrid operation suite, other (n=2)
